# Changes in the histological spectrum of glomerular diseases in the past 16 years in the North-Eastern region of Romania

**DOI:** 10.1186/1471-2369-14-148

**Published:** 2013-07-15

**Authors:** Carmen Volovăt, Irina Cãruntu, Camelia Costin, Alina Stefan, Raluca Popa, Simona Volovăt, Dimitrie Siriopol, Luminita Voroneanu, Ionut Nistor, Liviu Segall, Adrian Covic

**Affiliations:** 1 Department of Nephrology, University Hospital “Dr. C.I. Parhon”, Iasi, Romania; 2 Nephrology Department, Faculty of Medicine, University of Medicine and Pharmacy “Gr. T. Popa”, Iasi, Romania

**Keywords:** Glomerular disease, Glomerulonephritis, Kidney disease, Tubulointerstitial disease

## Abstract

**Background:**

The aim of this study was to describe the findings of renal biopsies from a large nephrology center in Iasi, Romania, performed between 2005 and 2010. We compared these findings with our previous ones, from 1995 to 2004, as well as with similar reports.

**Methods:**

We studied retrospectively 239 renal biopsies. The indications for renal biopsy were categorized into: nephrotic syndrome, acute nephritic syndrome, asymptomatic urinary abnormalities, acute kidney injury, and chronic kidney disease of unknown etiology.

**Results:**

During the past 16 years, a gradual increase in the annual number of renal biopsies/per million population (p.m.p.)/year was observed, although this incidence remained lower than in other European countries. Nephrotic syndrome was the indication for renal biopsy in over 50% of cases. Glomerulonephritis (GN) was the main histological diagnosis in 91% of cases, of which 56% were primary GN and 35% were secondary GN. The frequency of various types of primary GN was: membranoproliferative GN (MPGN) - 29.3%, membranous nephropathy (MN) -27.5%, focal segmental glomerulosclerosis (FSGS) - 17.2%, mesangial GN (including IgAN) -13.7%, crescentic GN - 9.4%, and minimal change disease (MCD) - 2.5%. Compared to the previously reported period (1994–2004), we observed a significant decrease in the frequency of MPGN and significant increases in the frequency of FSGS and, particularly MN - which more than doubled.

**Conclusion:**

We report significant changes in the histological spectrum of GN in North-Eastern Romania in 2005–2010, compared to the previously reported 10-yrs. These changes seem to be following a trend that has also been observed in Western countries a few decades ago, and which may have a socioeconomic explanation.

## Background

Histopathological diagnosis is a *sine qua non* prerequisite for the adequate management of most primary glomerular diseases in adults. National renal biopsy registries are available in Western European countries like France, Italy, Scotland, and Spain [1-6]. In contrast, in several Eastern European countries such registries are often lacking, incomplete or outdated [7]. However, the few existing reports suggest significant differences in the epidemiology of glomerulonephritis (GN) between Eastern and Western Europe, including, for example, a higher prevalence of IgA nephropathy (IgAN) and a lower prevalence of membranoproliferative glomerulonephritis (MPGN) in the latter region [8].

The aim of this study was to describe the findings of renal biopsies that were performed in a single large nephrology center in Iasi, covering the North-Eastern region of Romania (six counties, over 4.75 million inhabitants), between 2005 and 2010. Furthermore, we compared these findings with our previous ones in the same center, from 1995 to 2004 [8], as well as with similar reports from other countries.

## Methods

We studied retrospectively 239 renal biopsies, performed between 2005 and 2010 in the Nephrology Department of the “Dr. C. I. Parhon” Hospital in Iasi. Our unit is the referral nephrology center for Moldavia, the Eastern region of Romania, which includes eight counties and a population of over 4.75 million inhabitants. The study protocol was approved by the Ethics Committee of University Hospital “Dr. C.I. Parhon” (Iasi, Romania).

The percutaneous technique with ultrasound guidance, Tru-Cut 14-gauge needles and Bard Magnum® reusable core biopsy instrument (Bard Biopsy Systems®) was used for all biopsies [9,10]. Fresh biopsy cores were evaluated on the dissecting microscope. A small renal cortical tissue (2–4 mm in length) was separated for immunofluorescence study. The remaining biopsy specimen was fixed in formaline and B5 (mercury- based fixative) to be evaluated under light microscopy. Paraffin sections were prepared and stained with hematoxylin and eosin, periodic acid Schiff, Masson trichrome, Congo red, and Jones silver methenamine stains [11]. All the renal biopsies were examined by the same pathologist and evaluated with light microscopy and immunofluorescence (using Ig A and Ig G staining in all biopsies, and C3 staining in only 67 biopsies). Kidney transplant biopsies were excluded and there were no pediatric cases included in our study.

Histological diagnoses were classified as follows [6,12]:

1. Primary GN: IgA nephropathy (IgAN), crescentic GN (CGN) (defined as CGN not fulfilling the criteria for systemic disease), minimal change disease (MCD), mesangial GN other than IgAN (MesGN), membranous nephropathy (MN), focal segmental glomerulosclerosis (FSGS), and membranoproliferative GN (MPGN);

2. Secondary GN: lupus nephritis (LN), systemic vasculitis (VAS) (defined and classified according to the 1994 International Chapel Hill Consensus Conference on the Nomenclature of Systemic Vasculitides [13]), Goodpasture’s syndrome, amyloidosis, cryoglobulinaemic GN, acute poststreptococcal GN (PSGN), other post-infectious GN, diabetic nephropathy (DN), Alport’s syndrome and other hereditary GN, GN associated with liver disease, GN associated with malignancy;

3. Tubulointerstitial nephropathies (TIN), including acute TIN and chronic TIN;

4. Vascular nephropathies (VN): benign and malignant nephroangiosclerosis (NAS) and haemolytic uraemic syndrome (HUS);

5. Miscellaneous findings, including normal renal tissue, unclassified nephropathies, and global glomerulosclerosis.

Significant clinical data were gathered from the patients’ medical files, including the following: clinical diagnosis, serum creatinine (sCr), 24-hour proteinuria, presence of haematuria, and presence of arterial hypertension. Hypertension was defined as a blood pressure above 140 mm Hg for the systolic and/or 90 mm Hg for the diastolic and/or ongoing antihypertensive therapy. Complications of renal biopsy were also noted. Severe complications were defined as: severe renal bleeding requiring blood transfusions, acute kidney injury from obstruction with blood clots, urosepsis, and death [14,15].

Clinical syndromes were categorized into: nephrotic syndrome (NS), acute nephritic syndrome (ANS), asymptomatic urinary abnormalities (AUA), acute kidney injury (AKI), and chronic kidney disease (CKD). The NS was defined as the association of proteinuria >3.5 g/day/1.73 m^2^ with oedema, hypoproteinaemia, hypoalbuminaemia, and hyperlipidemia. Acute nephritic syndrome was defined as variable associations of at least three of the following manifestations: proteinuria, haematuria, hypertension, oedema, oliguria, and reduced glomerular filtration rate (GFR). The AUA encompassed isolated moderate proteinuria and/or microscopic haematuria. Acute kidney injury was defined as a sudden decrease in glomerular filtration rate (GFR), while CKD was diagnosed when low GFR (< 60 ml/min/1.73 m^2^) persisting for at least 3 months [16].

### Data analysis

The SPSS 16.0 statistics package was used (SPSS SystatInc, Chicago IL, USA). The annual incidence was defined as the number of new cases per year and per million populations (p.m.p.). Chi-square and Fisher’s exact tests were used to compare qualitative variables. P values <0.05 were considered statistically significant.

## Results

Between 1994 and 2010 a number of 559 renal biopsies were performed in our center, of which 320 from 1994 to 2004 (period 1) and 239 from 2005 to 2010 (period 2). Adequate samples of renal tissue were obtained in 514 (91.23%) biopsies, which underwent histological examination and were included in this report. The mean±standard deviation (SD) number of glomeruli per sample was 9.1±4.9.The incidence of renal biopsies increased from 10.7 per million population (p.m.p.)/year in 1994 to 11.5 p.m.p/year in 2004 and to 12.8 p.m.p/year in 2010. These figures are much lower than those reported by other European registries (e.g. 44.1–69.3 p.m.p./year in the Czech Republic [17], 48 p.m.p./year in Spain [18], and 16.3–20.1 p.m.p./year in France [5]), except for the Serbian registry where the renal biopsy rate is similar to ours (10.8 p.m.p./year [7]).

The patients’ mean age was 41.9±2.8 years-old and 58.5% were male. The distribution of clinical syndromes in relation with age and period of evaluation is presented in Table 1. Nephrotic syndrome was the most common indication for renal biopsy (53.4% of all cases). Other indications were AKI (15%), ANS and CKD (both with 14%) and AUA (3.6%).

**Table 1:** Differences in clinical presentation depending on period and age of patients

**Clinical syndrome**	**Whole population N= 559 (%)**	**Period 1 (1995–2004)N= 320 (%)**	**Period 2 (2005–2010) N= 239 (%)**	**<60 years N=435 (77.8%)**		**≥60years N=124 (22.18)**	
				**Period 1 N= 252 (%)**	**Period 2 N= 183 (%)**	**Period 1 N= 68 (%)**	**Period 2 N= 56 (%)**
Nephrotic syndrome	53.4	51.6	56	55.5*	60.6*	36.8	41
Acute nephritic syndrome	14	8.4	18.9	7.9	21.3	10.2	10.7
AUA	3.6	3.3	4.2	7.8	5.5*	2.9	0*
AKI	15	19.1	8.7	19.1	4.9*	19.2	21.5
CKD	14	17.6	12.2	12.7*	7.7*	30.9	26.8

*Significant difference between patients aged ≥60 vs those<60 years-old, (p <0.05).

The frequency of the main histological diagnoses is shown in Table 2, in comparison with those reported by the Italian (IRRB) [6], Czech (CRRB) [17] and Serbian (SRRB) [7] renal biopsy registries. Overall, primary GN was the most common diagnosis in our center (56%), as well as in the other three mentioned registries, followed by secondary GN (35%), TIN (2.5%), and VN (2.5%). The most common type of primary GN was MPGN (1.19 p.m.p./year), followed by MN (1.13 p.m.p./year), FSGS (0.70 p.m.p./year) and MesGN (including IgAN – 0.56 p.m.p./year)) (Table 3). However, a decrease in the frequency of MPGN was noted from 1994 (2.16 p.m.p./year) to 2010 (0.9 p.m.p./year). In contrast, the frequency of primary MN doubled between the two periods (from 0.51 p.m.p./year to 1.13 p.m.p/year). The frequencies of MN and FSGS were similar in our center and in the IRRB, whereas MesGN had a much lower percentage in our center than in IRRB and CRRB (16.6% *vs* 43.5% vs 45.8% and 37.3%, respectively). We found CGN in 11.2% of cases (0.37 p.m.p./year), compared to 2.3% in the IRRB, 3.2% in the CRRB and 5.1% in the SRRB.

**Table 2:** Distribution of histological diagnoses

	**Iasi, Romania**			**Italian Registry**[6]**] (N=14 607) % (p.m.p/year)**	**Czech Registry**[17]**] (N=4 004) % (p.m.p/year)**	**Serbian Registry**[7]**] (N=1 626) % (p.m.p/year)**
	**Period 1 (N=304) % (p.m.p/year)**	**Period 2 (N= 210) % (p.m.p/year)**	**Overall (N= 514) % (p.m.p/year)**			
**Primary GN**	56.6 (3.62)	55.3 (4.07)	56 (3.79)	70.8 (29.62)	59.8 (33.21)	64.0 (6.95)
**Secondary GN**	32.3 (2.06)	39.1 (2.88)	35.1 (2.36)	23.7 (11.38)	25.4 (14.11)	24.7 (2.68)
**TIN**	1.9 (0.12)	3.3 (0.24)	2.5 (0.17)	2.3 (2.44)	4.4 (2.44)	3.13 (0.34)
**VN**	3.6 (0.23)	0.9 (0.07)	2.5 (0.17)	3.2 (2.16)	3.4 (1.89)	4.42 (0.49)
**Miscellaneous**	5.6 (0.35)	1.4 (0.11)	3.9 (0.26)	N/A	2.4 (1.33)	N/A

Comparison with other European national registries of renal biopsy.

**TIN** - tubulointerstitial nephropathies; **VN** - vascular nephropathies.

Data are expressed in percentage and as renal biopsies /per million population (p.m.p.)/year.

**Table 3:** Incidence of different forms of primary glomerulonephritis

	**MPGN % (p.m.p/year)**	**MesGN % (p.m.p/year)**	**MN % (p.m.p/year)**	**CGN % (p.m.p/year)**	**FSGS % (p.m.p/year)**	**MCD % (p.m.p/year)**
**Iasi, Romania (1995–2010)**						
Period 1, N=172	38.4 (1.39)	18.6 (0.67)	13.9 (0.51)	12.2 (0.44)	11.1 (0.40)	5.8 (0.21)
Period 2, N=116	29.3^a^ (1.19)	13.8 (0.56)	27.6^a^ (1.13)	9.5 (0.37)	17.2 (0.70)	2.6 (0.11)
Overall N=288	34.7^b^ (1.31)	16.6^b^ (0.63)	19.5^b^ (0.74)	11.2^b^ (0.42)	13.5 (0.51)	4.5 (0.17)
**IRRB**[6], **N**=**6990**	6.6 (1.62)	43.5 (10.67)	23.4 (5.73)	2.3 (0.57)	13.1 (3.23)	9.2 (2.25)
**CRRB**[17], **N**= **2333**	4.6 (1.49)	45.8 (14.82)	9.3 (3.01)	3.2 (1.04)	10.8 (3.37)	12.5 (4.04)
**SRRB**[7], **N**=**955**; <**60 years** (%)	10.0	37.3	18.9	5.1	18.9	7.8
**SRRB**[7], **N**=**87**; ≥**60 years** (%)	16.1	24.0	28.7	6.9	18.4	4.6
**SRRB**[7], **N**=**1042**; **all ages** (**renal biopsies**/**per million population** (**p**.**m**.**p**.)/**year**)	0.73	2.51	1.37	0.37	1.31	0.53

**CGN** - crescentic glomerulonephritis); **FSGS** - focal segmental glomerulosclerosis; **MCD** - minimal change disease; **MesGN** - mesangioproliferative glomerulonephritis (including IgAN); **MN** - membranous nephropathy; **MPGN** - membranoproliferative glomerulonephritis. **IRRB** - Italian Registry of Renal Biopsies; **CRRB** - Czech Registry of Renal Biopsies; **SRRB** - Serbian Registry of Renal Biopsies.

^a^significant differences between period 1 and period 2 (p<0.05).

^b^significant differences between Iasi vs. IRRB vs. CRRB vs. SRRB (p<0.05).

Data are expressed in percentage and as renal biopsies /per million population (p.m.p.)/year.

**Figure 1:**
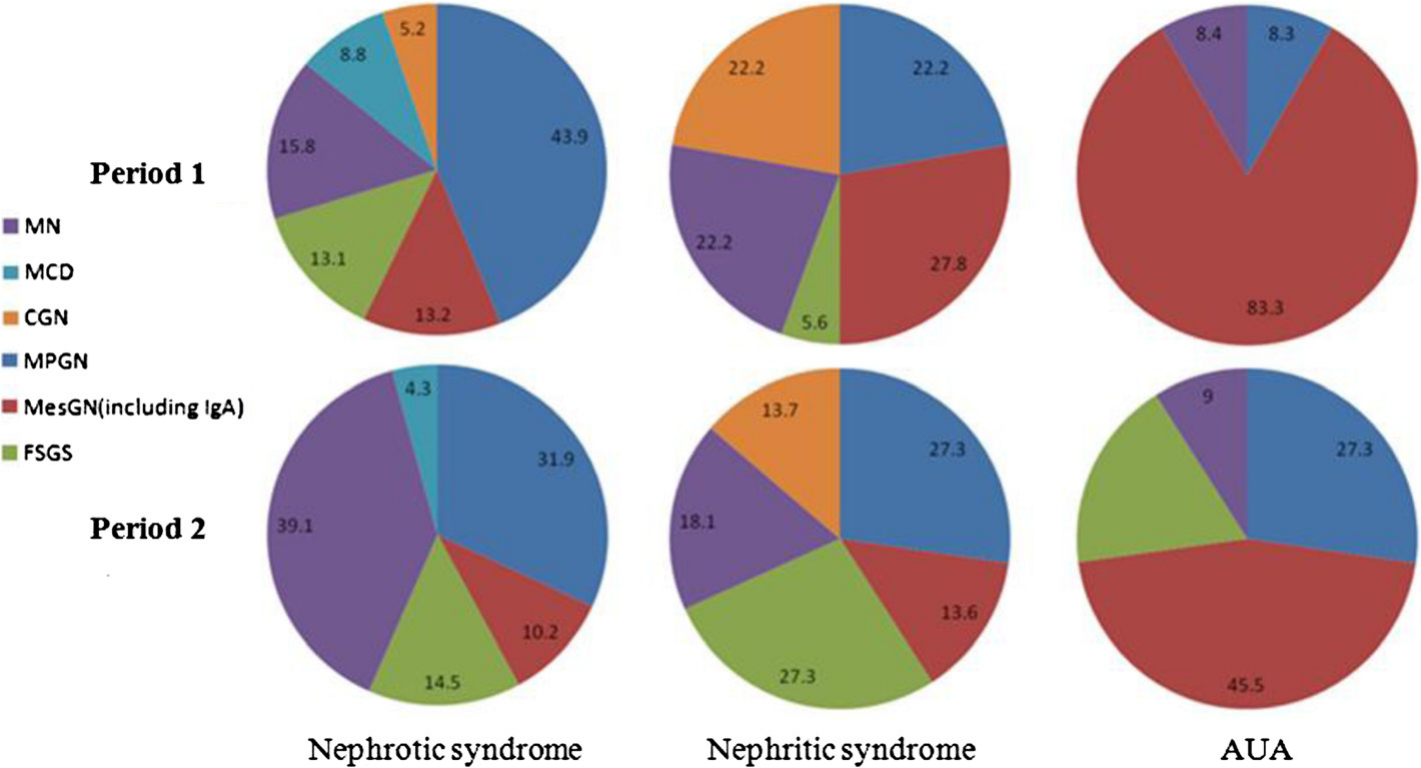
**Correlations between histological diagnoses and clinical presentations in primary glomerulonephritis. CGN** - crescentic glomerulonephritis); **FSGS** - focal segmental glomerulosclerosis; **MCD** - minimal change disease; **MesGN** - mesangioproliferative glomerulonephritis (including IgAN); **MN** - membranous nephropathy; **MPGN** - membranoproliferative glomerulonephritis.

Correlations between histological and clinical findings are shown in Table 4 and Figure 1. The GN most often underlying the NS in the two studied periods were MPGN (43.9% and 31.9%, respectively) and MN (15.8% and 39.1%, respectively), but with a significant increase for MN from period 1 (0.59 p.m.p./year) to period 2 (1.63 p.m.p./year) (P = 0.005). ANS was mainly caused by MesGN (including IgAN) in period 1 and by MPGN in period 2. The main cause of AUA was MesGN (including IgAN) in both periods. Among secondary GN, the NS was most frequently due to immune-mediated GN (including LN and VAS) (41% - 0.56 p.m.p./year) and infectious disease-associated GN (33% - 0.46 p.m.p./year). ANS was mainly caused by immune-mediated GN in the both periods (55.2% and 54.4%) (Table 4).

**Table 4:** Correlations between histological diagnoses and clinical presentations

	**Nephrotic syndrome (%)**		**Nephritic syndrome (%)**		**AUA (%)**	
	**Period 1**	**Period 2**	**Period 1**	**Period 2**	**Period 1**	**Period 2**
**Primary GN**						
MPGN	43.9	31.9	22.2	27.3	8.3	27.3
MesGN(including IgA)	13.2^a^	10.2	27.8	13.6	83.3^d^	45.5
FSGS	13.1	14.5	5.6^d^	27.3	0^d^	18.2
MN	15.8^d^	39.1	22.2	18.1	8.4	9
MCD	8.8^c^	4.3	0	0	0	0
CGN	5.2	0	22.2	13.7	0	0
*Total* (*N*)	*114*	*69*	*18*	*22*	*12*	*11*
**Secondary GN**						
Immune-mediated	41.1	41	11.1^d^	54.4	50	50
Dysgammaglobulinaemia associated GN	21.6	20.5	0	18.2	50^d^	0
Infectious disease associated GN	33.3	33.4^c^	88.9^d^	27.3	0^b^	50
Others (metabolic, hereditary, etc.)	4	5.1	0	0	0	0
*Total* (*N*)	*51*	*39*	*91*	*1*	*2*	*2*

**CGN** - crescentic glomerulonephritis); **FSGS** - focal segmental glomerulosclerosis; **MCD** - minimal change disease; **MesGN** - mesangioproliferative glomerulonephritis (including IgAN); **MN** - membranous nephropathy; **MPGN** - membranoproliferative glomerulonephritis.

^a^Significantly different from nephrotic syndrome and AUA.

^b^Significantly different from nephritic syndrome and AUA.

^c^Significantly different from nephrotic and nephritic syndrome.

^d^Significant differences between the 2 periods studied.

We found no statistical difference in the proteinuria level between different types of primary GN. By comparison, the serum creatinine was highest in the CGN group (3.00±3.60 mg/dl) and lowest in the MCD (0.94±0.39) (Table 5).

**Table 5:** Serum creatinine and proteinuria in primary glomerulonephritis

	**MPGN**	**MesGN**	**MN**	**CGN**	**FSGS**	**MCD**
Serum creatinine, mg/dl	1.63±1.60	2.27±2.24	2.08±2.89	3.00±3.06^a^	2.42±3.17	0.94±0.39
Proteinuria, g/day	8.44±5.71	6.75±8.49	9.11±6.99	5.77±5.41	5.57±6.92	1.33±5.76

Data are expressed as mean±SD. **CGN** - crescentic glomerulonephritis; **FSGS** - focal segmental glomerulosclerosis; **MCD** - minimal change disease; **MesGN** - mesangioproliferative glomerulonephritis (including IgAN); **MN** - membranous nephropathy; **MPGN** - membranoproliferative glomerulonephritis.

^a^Significant differences between CGN and MCD (p<0.05).

Immune-mediated GN (58.3%) and dysgammaglobulinaemia-associated GN (i.e. myeloma and amyloidosis, 13.8%) had similar percentages in our center, in the IRRB, and in the CRRB but lower than the SRRB with 90% on the patients below 60 years old. The frequency of LN increased from 25.5% (0.48 p.m.p./year) to 28% (0.56 p.m.p./year), whereas that of VAS remained constant across periods (27.5% or 0.53 p.m.p./year in the first period compared to 21.9% or 0.56 p.m.p./year). The incidence of infectious disease-associated GN also remained constant (25.5% or 0.53 p.m.p./years in the first period vs. 21% or 0.63 p.m.p./year in the second one), but, by comparison with the aforementioned registries, it was found in a larger proportion (23.8% of the secondary GN vs 3% in the IRRB). DN and hereditary GN were less frequent and with a decreasing percentage (Table 6).

**Table 6:** Incidence of different types of secondary glomerulonephritis

	**Iasi, Romania**			**IRRB**[6]***N = 2348***	**CRRB**[17]***N = 990***	**SRRB**[7], **N** = **373**; <**60 years** (%)	**SRRB**[7], **N** = **29**; ≥**60 years** (%)	**SRRB**[7], **N** = **402**; **all ages** (**p**.**m**.**p**/**year**)
	**Period 1 *N = 98***	**Period 2 *N = 82***	**Overall *N = 180***					
**Immune**-**mediated GN**	60.2 (1.24)	56 (1.61)	58.3 (1.38)	57.6 (4.68)	48.6 (6.67)	90.1	N/A	2.33
Necrotizing vasculitides	27.5 (0.53)	21.9 (0.56)	25 (0.54)		15.6 (2.14)	13.7	31.0	0.40
Lupus nephritis	25.5 (0.48)	28 (0.56)	26.6 (0.51)		23 (3.16)	75.6	17.2	1.91
Others (Goodpasture’s syndrome, rheumatoid arthritis, etc.)	7.1 (0.15)	6.1 (0.17)	6.6 (0.16)		6.9 (0.94)	0.8	N/A	0.02
**Dysgammaglobulinemia**-**associated GN**	12.2 (0.25)	15.8 (0.46)	13.8 (0.33)	15.9 (1.31)	14.9 (2.05)	7.2	41.4	0.26
Renal amyloidosis	10.2 (0.21)	9.7 (0.29)	10 (0.24)		9.9 (1.35)	6.4	31.0	0.22
Cryoglobulinemia	2 (0.04)	6 (0.17)	3.8 (0.09)		5 (0.14)	0.8	10.4	0.04
**Infectious diseases associated GN**	25.5 (0.53)	21.9 (0.63)	23.8 (0.57)	3 (0.23)	N/A	N/A	N/A	N/A
**Others (metabolic, hereditary, etc.)**	2.9 (0.04)	6 (0.17)	3.8 (0.09)	23.5 (1.16)	36.5 (5.01)	N/A	N/A	N/A

Data are expressed in percentage and as renal biopsies /per million population (p.m.p.)/year.

**IRRB** - Italian Registry of Renal Biopsies; **CRRB** - Czech Registry of Renal Biopsies; **SRRB** - Serbian Registry of Renal Biopsies.

Macroscopic hematuria was the most frequent complication of renal biopsies, encountered in 5.2% of patients. Blood transfusions were administered in only 0.9% of cases, but no surgical or radiological interventions for bleeding were necessary.

## Discussion

This study provides comprehensive information about the spectrum of biopsy-proven GN in the past 16 years in our center. Additionally, we compared our findings to those reported by the Italian, the Serbian and the Czech renal biopsy registries. The Italian registry is representative for the Western Europe and the Czech and Serbian registries much more related, from the geographical and economical point of view, with our report. The biopsy frequency is significantly lower than that of the mentioned registries, and that could be explained by the lower addressability to health care providers, at least in initial, non-symptomatic disease. This conservative approach could also be a consequence of the opinion of a part of the physicians to perform a biopsy only when they felt that the pathology could alter the therapy or when patients had signs of progressive renal disease.

The main findings of our study, in comparison with other European reports are: (a) a similar frequency but with a lower incidence of primary and secondary GN, (b) a higher frequency of MPGN, but with a clear descending trend over the past 16 years, (c) a lower incidence of primary MN, but with an ascending trend over the past 16 years, (d) a higher and steady frequency of infectious disease-associated GN.

Glomerulonephritis was the main diagnosis in the vast majority of our biopsies (91%), which is similar to the reports of Schena et al. [19], Verde et al. [2], and Naumovic et al. [7].

The most frequent clinical presentation was NS, followed by AKI, ANS, and AUA. These data were in contrast with the reports from Western Europe, where the most common clinical indications for kidney biopsy were AUA [1,17]. However, when comparing the data from 1995–2004 with those from 2005–2010, we found a similar trend as in the Western registries [18,19], with less patients having AKI [19.06% *vs* 8.7%]. Similarly to other reports [18,19], NS was seen in about 10% of FSGS and 25% of MN cases. Significant differences from all other reports were found for MPGN and MCD [18-21].

For the last 16 years, MPGN (primary and secondary) has remained the first cause of NS in North-Eastern Romania. This feature has been described in other centers in Romania, as well [8]. A possible explanation might be the high prevalence of infectious diseases in our country, such as viral B and C hepatitis and streptococcal infections [22,23]. However, from 1995 to 2010, the incidence of MPGN in our center has continuously declined. Such a trend was previously reported in countries like France, where a decline in the incidence of rheumatic fever was associated with a parallel decline in the incidence of MPGN and post-streptococcal GN [5]. It seems that the epidemiology of MPGN is strongly related to socio-economic conditions and that improvements in incomes, social and health care status are associated with a decrease in the incidence of MPGN [24]. These advances have been significant in Romania after 1989, as well as in other Eastern European countries [8,24].

According to our data, MN was the second cause of NS, followed by FSGS and MesGN (including IgAN). The increasing prevalence of FSGS in our center is comparable to that seen in previous decades in the USA, where FSGS has now become the most common cause of NS [25,26]. On the other hand, in our study MesGN was the first cause of AUA.

Acute kidney injury was the most common clinical presentation of patients with VAS and CGN. We found that 11.1% of primary GN were CGN and almost one-third of all cases with AKI had CGN. These data are similar to the Italian registry [4], where CGN were found in 34.1% of cases presenting with AKI.

Our study has several limitations. We analyzed retrospectively only patients from a single centre, but the “Dr. C.I. Parhon” University Hospital is the only reference centre for the entire North-Eastern region of Romania (six counties, over 4.75 million inhabitants). The economy of this region is mainly agricultural with the regional gross domestic product per capita being the lowest in Romania (€ 7 200 in 2008), at about two-thirds of the national average and this could explain the lower addressability to health care providers, at least in initial, non-symptomatic disease. Corroborated with a conservative approach, this lead to a lower biopsy frequency with a relatively low number of patients included in our study. Finally, although we performed immunohistology in all biopsies, the C3 staining was available only from 2009, thus preventing the analysis of different subtypes of GNMP.

## Conclusions

Nephrotic syndrome was the indication for renal biopsy in more than 50% of cases in our center. Membranoproliferative GN has remained the most common type of primary GN, whereas immune-mediated diseases (LN and VAS) were the main causes of secondary GN. Serious complications of renal biopsy were rare.

We believe that our data represent an important contribution to the epidemiology of kidney diseases in Europe.

## Competing interests

The authors declare that they have no competing interests.

## Authors’ contributions

CV: researched and wrote and revised the manuscript. IC: researched and revised the manuscript. CC: researched and revised the manuscript. AS: researched and revised the manuscript. RP: researched and revised the manuscript. SV: researched and revised the manuscript. DS: researched, wrote and revised the manuscript. LV: researched, wrote and revised the manuscript. IN: researched, wrote and revised the manuscript. LS: researched, wrote and revised the manuscript. AC: conceived the idea for the paper and revised the manuscript. All authors read and approved the final manuscript.

## Pre-publication history

The pre-publication history for this paper can be accessed here:

http://www.biomedcentral.com/1471-2369/14/148/prepub
